# 5-Aza-2′-deoxycytidine Leads to Reduced Embryo Implantation and Reduced Expression of DNA Methyltransferases and Essential Endometrial Genes

**DOI:** 10.1371/journal.pone.0045364

**Published:** 2012-09-28

**Authors:** Yu-Bin Ding, Chun-Lan Long, Xue-Qing Liu, Xue-Mei Chen, Liang-Rui Guo, Yin-Yin Xia, Jun-Lin He, Ying-Xiong Wang

**Affiliations:** Department of Reproductive Biology, Chongqing Medical University, Chongqing, People’s Republic of China; Florida International University, United States of America

## Abstract

**Background:**

The DNA demethylating agent 5-aza-2′-deoxycytidine (5-aza-CdR) incorporates into DNA and decreases DNA methylation, sparking interest in its use as a potential therapeutic agent. We aimed to determine the effects of maternal 5-aza-CdR treatment on embryo implantation in the mouse and to evaluate whether these effects are associated with decreased levels of DNA methyltransferases (Dnmts) and three genes (*estrogen receptor α* [*Esr1*], *progesterone receptor* [*Pgr*], and *homeobox A10* [*Hoxa10*]) that are vital for control of endometrial changes during implantation.

**Methods and Principal Findings:**

Mice treated with 5-aza-CdR had a dose-dependent decrease in number of implantation sites, with defected endometrial decidualization and stromal cell proliferation. Western blot analysis on pseudo-pregnant day 3 (PD3) showed that 0.1 mg/kg 5-aza-CdR significantly repressed Dnmt3a protein level, and 0.5 mg/kg 5-aza-CdR significantly repressed Dnmt1, Dnmt3a, and Dnmt3b protein levels in the endometrium. On PD5, mice showed significantly decreased Dnmt3a protein level with 0.1 mg/kg 5-aza-CdR, and significantly decreased Dnmt1 and Dnmt3a with 0.5 mg/kg 5-aza-CdR. Immunohistochemical staining showed that 5-aza-CdR repressed DNMT expression in a cell type–specific fashion within the uterus, including decreased expression of Dnmt1 in luminal and/or glandular epithelium and of Dnmt3a and Dnmt3b in stroma. Furthermore, the 5′ flanking regions of the *Esr1*, *Pgr*, and *Hoxa10* were hypomethylated on PD5. Interestingly, the higher (0.5 mg/kg) dose of 5-aza-CdR decreased protein expression of Esr1, Pgr, and Hoxa10 in the endometrium on PD5 in both methylation-dependent and methylation-independent manners.

**Conclusions:**

The effects of 5-aza-CdR on embryo implantation in mice were associated with altered expression of endometrial Dnmts and genes controlling endometrial changes, suggesting that altered gene methylation, and not cytotoxicity alone, contributes to implantation defects induced by 5-aza-CdR.

## Introduction

The nucleoside analog 5-aza-2′-deoxycytidine (5-aza-CdR) is a potent DNA demethylating agent that has been widely used to demonstrate the correlation between demethylation and reactivation of specific genes [1]. 5-Aza-CdR induces cell cycle arrest, cell differentiation, and cell death mainly by inhibiting post-replication methylation of DNA. In mice, exposure to 5-aza-CdR during development alters gene expression, causes malformations, and suppresses growth; administration of 5-aza-CdR to pregnant mice or rats at mid- or late-gestational periods elicits multiple characteristic defects [2]–[4].

Among the DNA cytosine-5-methyltransferase (Dnmt) family of DNA methylases, three members (Dnmt1, 3a, and 3b) have been shown to mediate the cytotoxic effects of 5-aza-CdR in mammals [5], [6]. 5-Aza-CdR inhibits DNMT and demethylates DNA by incorporation into DNA [1], degradation of DNMT [7], downregulation of DNMT mRNA and protein levels [8]–[11], or repression of DNMT enzymatic activity [11], [12], leading to changes in gene reactivation. 5-Aza-CdR also downregulates gene expression independently of DNA methylation [9], [13]–[17].

**Figure 1:**
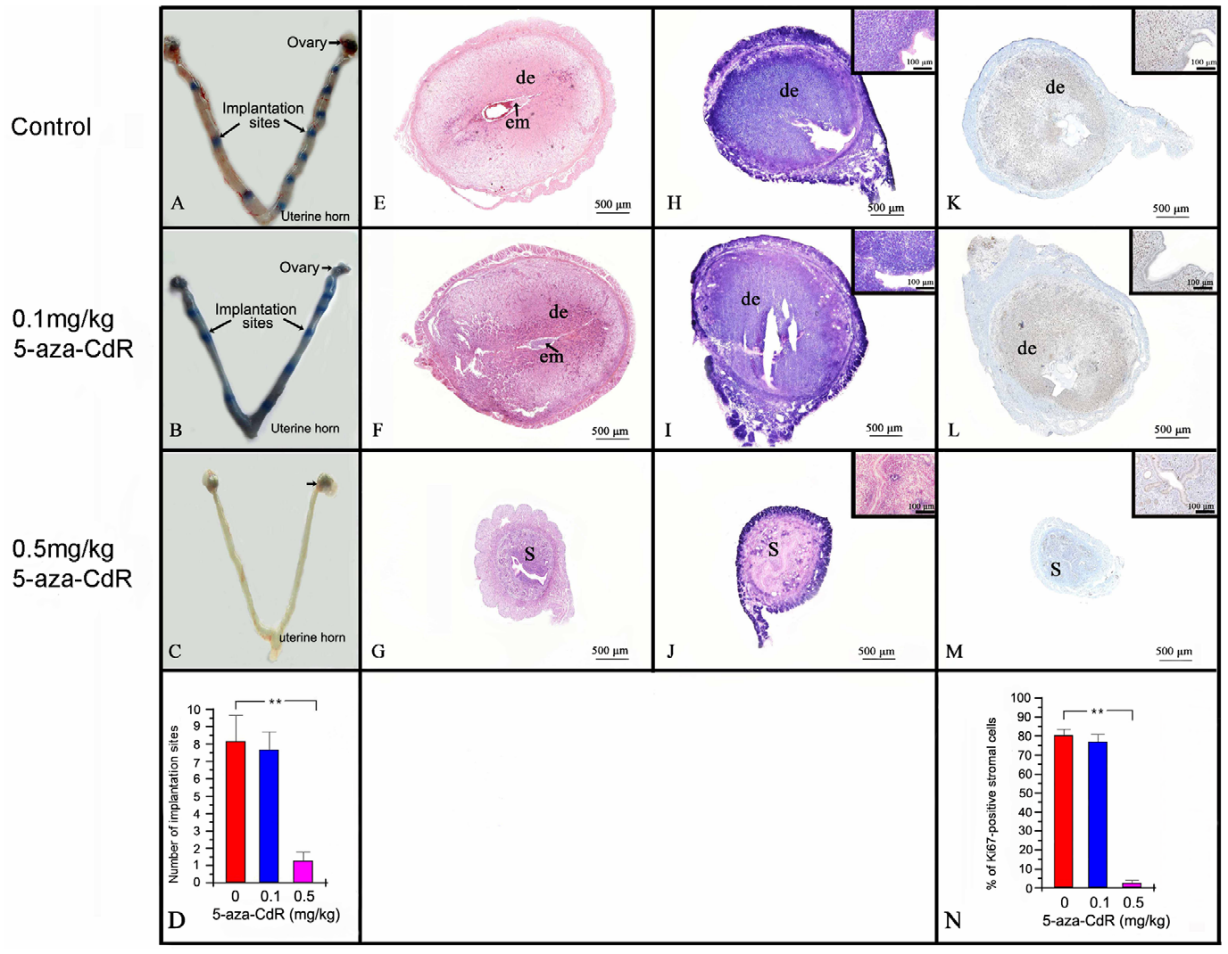
Effects of 5-aza-CdR on embryo implantation, cell proliferation, and decidualization. (A–C) Embryo implantation sites (blue dots with arrows) in mice treated with or without 5-aza-CdR, stained by intravenous Chicago Blue B injection on day 5 of pregnancy (D5). (D) Graph showing the number of implantation sites in each group (*n* = 6 for each group). Bars indicate the mean±S.D. **p<0.001. (E–G) Hematoxylin and eosin staining of longitudinal uterine sections on D7 after 4 days treatment with 5-aza-CdR, indicates defected development of uterus during post-implantation period in mice treated by 0.5 mg/kg 5-aza-CdR. de, decidua; em, embryo; S, stroma. (H–J) Endometrial decidualization as indicated by alkaline phosphatase activity (blue) on PD6 in oil-induced decidulized uteruses (red indicates negative staining). (K–M) Endometrial stromal cell proliferation on PD6 in oil-induced decidualized uteruses, indicated by immunostaining with rabbit anti-Ki67 (brown). (N) Graph showing the number of Ki67-positive stromal cells in each group (*n = *6 for each group). Bars indicate the mean±S.D. **p<0.001.

Embryo implantation is a critical step in embryo development and pregnancy outcome. To enable implantation, the uterus goes through changes that prepare it to receive the embryo. Recent studies have suggested that DNA methylation may be involved in endometrial change during periimplantation stages. For instance, mRNA levels of *Dnmt1*, *Dnmt3a*, and *Dnmt3b* are altered according to the phase of the menstrual cycle [18], [19]. Several genes are regulated by DNA methylation in relevant cell types, including *estrogen receptor 2* (*ESR2*) [20], *paired box gene 2* (*PAX2*) [21], *E-cadherin* [22], and *S100A4* [23] in endometrial cells, and *killer cell immunoglobulin-like receptor 3DL3* (*KIR3DL3*) in natural killer cells [24]. In addition, human endometrial stromal cells treated with 5-aza-CdR showed significantly enhanced expression of 76 genes by microarray analysis, including two established decidualization marker genes. These expression patterns are similar to those of cells treated with medroxyprogesterone acetate [25].

**Figure 2:**
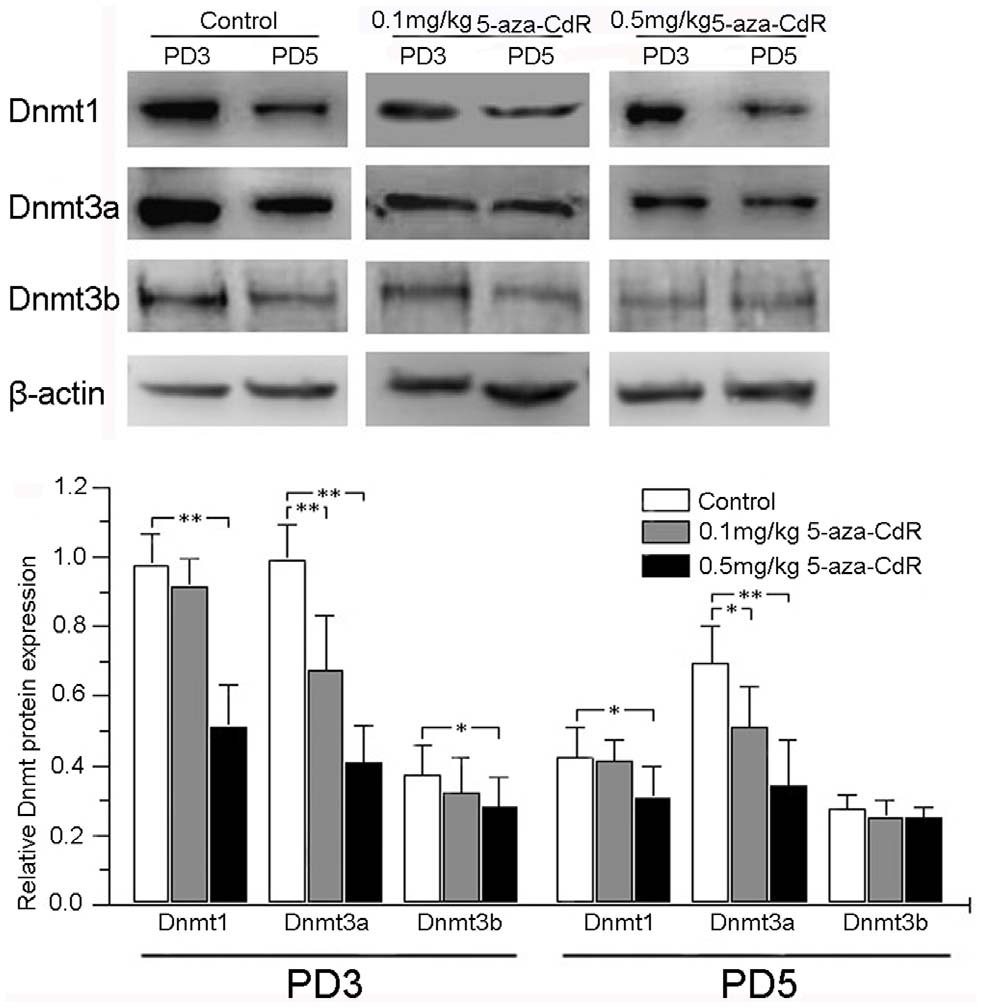
Effects of 5-aza-CdR on Dnmt1, Dnmt3a, and Dnmt3b expression in mouse endometrium by western blotting. Mice were treated for 2 or 4 days with 0 (control), 0.1, or 0.5 mg/kg 5-aza-CdR, and tissues were collected for analysis on PD3 or PD5. β-actin was assessed as a loading control. *p<0.01; **p<0.001.

Currently, the effect of 5-aza-CdR on mouse endometrial gene expression, DNA methylation, and embryo implantation from the beginning of fertilization is unknown. Here, we investigated whether the effect of 5-aza-CdR on embryo implantation in mice was associated with endometrial DNMT expression. We also investigated the effect of 5-aza-CdR on methylation of the flanking regions of *estrogen receptor α* (*Esr1*), *progesterone receptor* (*Pgr*), and *homeobox A10* (*Hoxa10*), which have key roles in implantation, and examined expression of their encoded proteins in mouse endometrium [26], [27] following 2 or 4 days of 5-aza-CdR administration.

## Results

### 5-Aza-CdR Reduced the Number of Implantation Sites and Impaired Decidualization, and Stromal Cell Proliferation

On D5 (after 4 days of treatment), the number of embryo implantation sites did not differ significantly between control mice and those treated with 0.1 mg/kg 5-aza-CdR (*p* = 0.249), but was greatly reduced in mice treated with 0.5 mg/kg 5-aza-CdR (*p*<0.001; Figure 1A–D). On D5, there were no differences in embryonic appearance or weight between the three treatment groups (data not shown). Hematoxylin and eosin staining of longitudinal uterine sections on D7 after 4 days treatment with 5-aza-CdR, indicates defected development of uterus during post-implantation period in mice treated by 0.5 mg/kg 5-aza-CdR (Figure 1E–G). Alkaline phosphatase activity is an indicator of stromal cell differentiation in response to decidualization [28], which is a vital step during implantation [29]. The oil-induced decidualized endometria on PD6 of mice treated with 0.1 mg/kg 5-aza-CdR for 4 days showed strong alkaline phosphatase activity, whereas the endometria of mice treated at 0.5 mg/kg had no detectable alkaline phosphatase activity (Figure 1H–J). In addition, an increase in the presence of Ki67-positive cells on PD6 indicated a decrease in proliferation of endometrial stromal cells in mice treated at 0.5 mg/kg for 4 days; there was no significant difference in the number of Ki67-positive cells in endometrial stroma of control mice versus mice treated at 0.1 mg/kg (Figure 1K–N).

**Figure 3:**
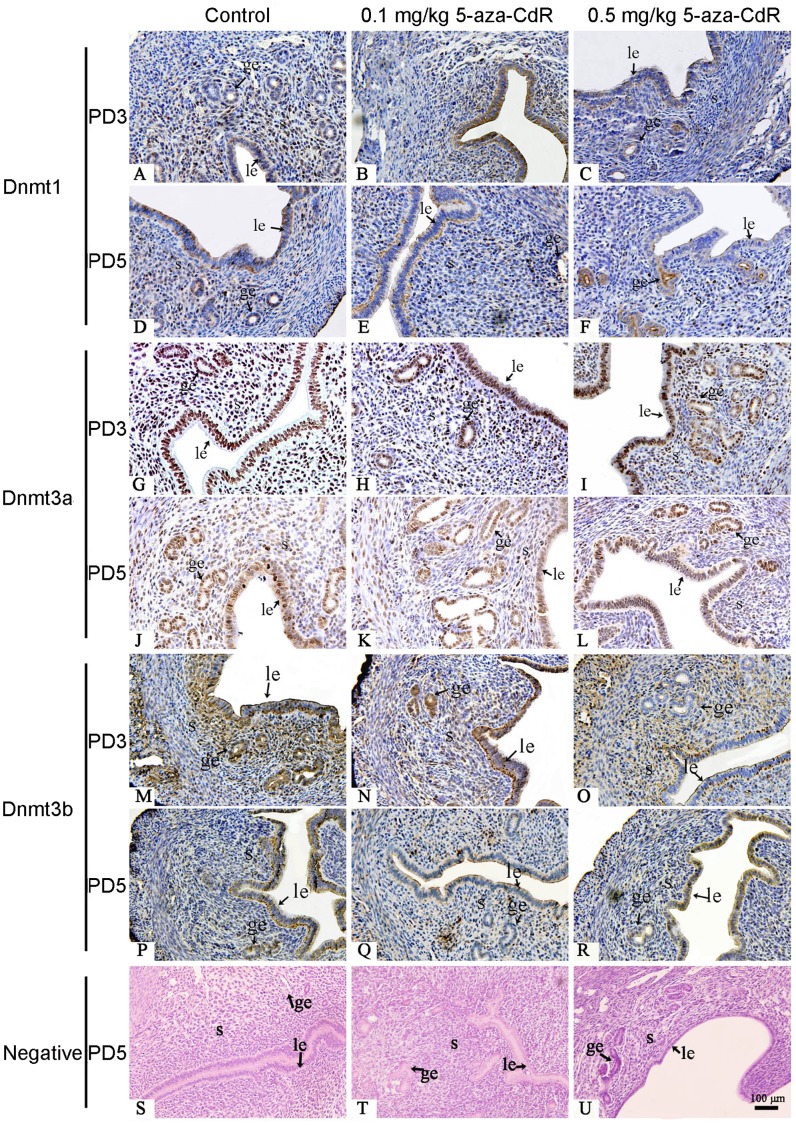
Effects of 5-aza-CdR on Dnmt1, Dnmt3a, and Dnmt3b expression in mouse endometrium by immunohistochemistry. Mice were treated for 2 or 4 days with 0 (control), 0.1, or 0.5 mg/kg 5-aza-CdR, and sections were prepared on PD3 or PD5. Immunoreactivity (brown) of Dnmt1 (A–F), Dnmt3a (G–L), and Dnmt3b (M–R) is shown. Sections that were not exposed to the primary antibody were used as negative controls (S–U) and stained with Hematoxylin and eosin. le, luminal epithelium; ge, glandular epithelium; S, stroma.

### 5-Aza-CdR Repressed Dnmt Protein Expression

Because 5-aza-CdR is an inhibitor of DNA methyltransferases, we analyzed Dnmt1, Dnmt3a, and Dnmt3b protein expression in mouse endometrium after 2 days (PD3) and 4 days (PD5) of treatment with 5-aza-CdR. On PD3, western blot analysis showed that 0.1 mg/kg 5-aza-CdR significantly repressed Dnmt3a expression (*p*<0.001) compared to the control, but did not significantly alter Dnmt1 (*p* = 0.129) or Dnmt3b (*p* = 0.167) expression. In contrast, 0.5 mg/kg 5-aza-CdR significantly repressed all three Dnmts (*p*<0.01 each). On PD5, the 0.1 mg/kg dose led to a decrease only in Dnmt3a protein (*p*<0.01), and the 0.5 mg/kg dose repressed Dnmt1 (*p*<0.01) and Dnmt3a (*p*<0.001), but Dnmt3b was not significantly repressed at either dose (*p* = 0.154 at 0.1 mg/kg; *p* = 0.105 at 0.5 mg/kg; Figure 2).

We next examined tissue sections to determine whether 5-aza-CdR affected Dnmt expression in a cell type–specific fashion within the uterus. On PD3, mice treated at 0.1 and 0.5 mg/kg showed a repressed Dnmt1 protein level in stroma compared to control mice, with no changes in the luminal or glandular epithelium (Figure 3A–C). On PD5, mice showed a decreased Dnmt1 protein level in stroma at 0.1 and 0.5 mg/kg 5-aza-CdR, and repressed expression in luminal epithelium at 0.5 mg/kg (Figure 3D–F). On PD3 and PD5, mice showed reduced expression of Dnmt3a in stroma, but not in luminal or glandular epithelium, at 0.1 and 0.5 mg/kg 5-aza-CdR (Figure 3G–L). On PD3, mice showed no change in Dnmt3b protein level in stroma at 0.1 or 0.5 mg/kg 5-aza-CdR, but showed decreased expression in luminal and glandular epithelia at 0.5 mg/kg (Figure 3M–O). On PD5, Dnmt3b protein levels were unchanged in stroma, luminal epithelia, and glandular epithelia at both doses (Figure 3P–R). These results are summarized in Table 1.

**Table 1:** Localization of DNA methyltransferases (Dnmts) in mouse endometrium following treatment with 5-aza-2′-deoxycytidine.

Protein	5-aza-CdR (mg/kg)	Day of pseudo-pregnancy	Stroma	Luminal epithelium	Glandular epithelium
**Dnmt1**	0	PD3	++	+	+
		PD5	+	+	+
	0.1	PD3	+	+	+
		PD5	−	+	+
	0.5	PD3	−	+	+
		PD5	−	−	+
**Dnmt3a**	0	PD3	+++	+++	+++
		PD5	++	+++	+++
	0.1	PD3	++	+++	+++
		PD5	+	+++	+++
	0.5	PD3	+	+++	+++
		PD5	+	+++	+++
**Dnmt3b**	0	PD3	++	++	++
		PD5	−	+	+
	0.1	PD3	++	++	++
		PD5	−	+	+
	0.5	PD3	++	+	+
		PD5	−	+	+

−, no staining; +, weak staining; ++, moderate staining; and +++, strong staining.

### 5-Aza-CdR Reduced Methylation of Hoxa10, a Gene that Controls Endometrial Change

**Figure 4:**
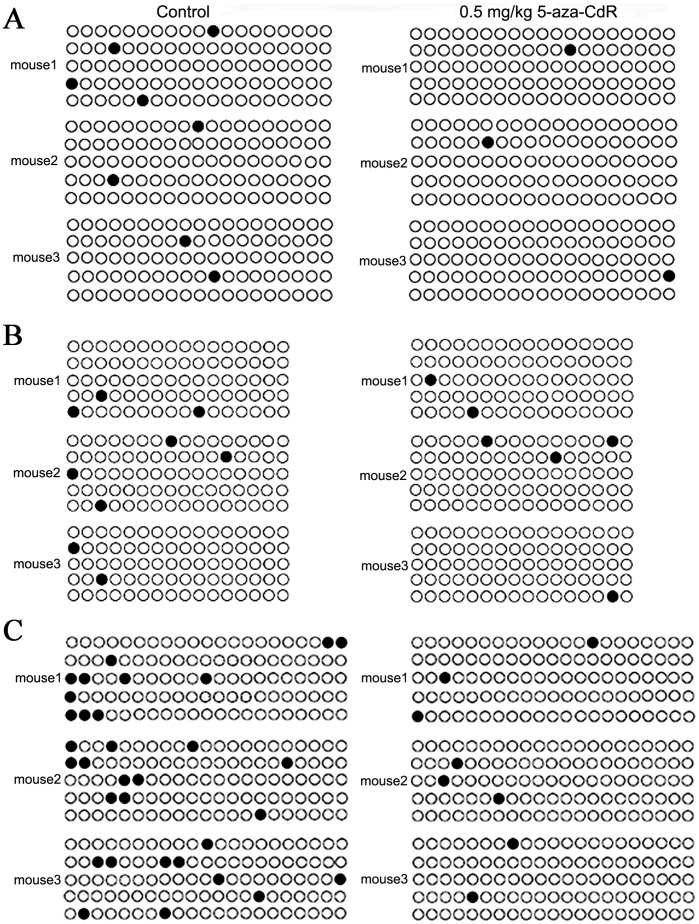
Effects of 5-aza-CdR on DNA methylation of genes controlling implantation in endometrial tissues. (A) *Esr1* promoter and 5′-UTR (exon 1) containing 19 CpG sites. (B) 5′-UTR (exon 1) of *Pgr* containing 16 CpG sites. (C) *Hoxa10* promoter and 5′-UTR (exon 1) containing 21 CpG sites. Each row of circles represents a single cloned allele (five clones per mouse). Each circle represents a single CpG site. Filled and open circles indicate methylated and unmethylated cytosines, respectively.

To investigate whether a 5-aza-CdR affects methylation in flanking regions of genes that are vital for endometrial changes in mouse embryo implantation, we used BSP to quantitatively assess the methylation status of each CpG site in the flanking regions of three genes: *estrogen receptor α* (*Esr1*), *progesterone receptor* (*Pgr*), and *homeobox A10* (*Hoxa10*). For each group (control and 0.5 mg/kg 5-aza-CdR), we randomly selected five clones of each gene region from each of three mice, resulting in 15 BSP analyses for each gene (Figure 4A–C). For *Esr1* (Figure 4A), there were 19 CpG sites spanning –73 to +327 nt of the promoter and 5′-UTR (exon 1). For *Pgr* (Figure 4B), there were 16 CpG sites spanning +119 to +396 nt of the 5′-UTR (exon 1). For *Hoxa10*, there were 21 CpG sites spanning –386 to +8 nt of the promoter and 5′-UTR (exon 1). The percentages of total methylated CpG sites in *Esr1*, *Pgr*, and *Hoxa10* in control mice were 2.807%, 3.750%, and 10.159%, respectively, indicating the baseline hypomethylation status of these regions. Compared to these controls, 0.5 mg/kg 5-aza-CdR significantly reduced methylation of the *Hoxa10* region (2.540%, *p*<0.001), but had no significant effect on *Esr1* (1.053%, *p* = 0.128) or *Pgr* (2.500%, *p* = 0.431).

### 5-Aza-CdR Decreased Expression of Proteins that Control Endometrial Change

**Figure 5:**
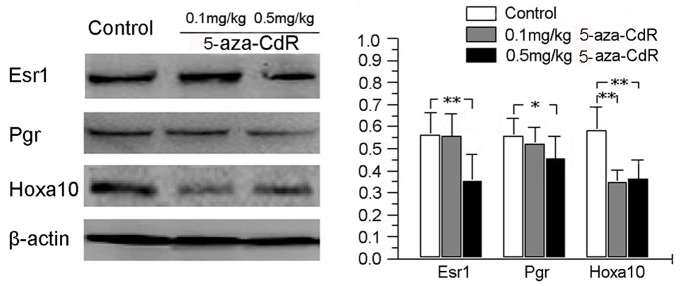
Effects of 5-aza-CdR on Esr1, Pgr, and Hoxa10 protein levels in mouse endometrium. Mice were treated with 0 (control), 0.1, or 0.5 mg/kg 5-aza-CdR for 4 days, and endometrium was analyzed by western blotting on PD5. β-actin was assessed as a loading control. *p<0.01; **p<0.001.

**Figure 6:**
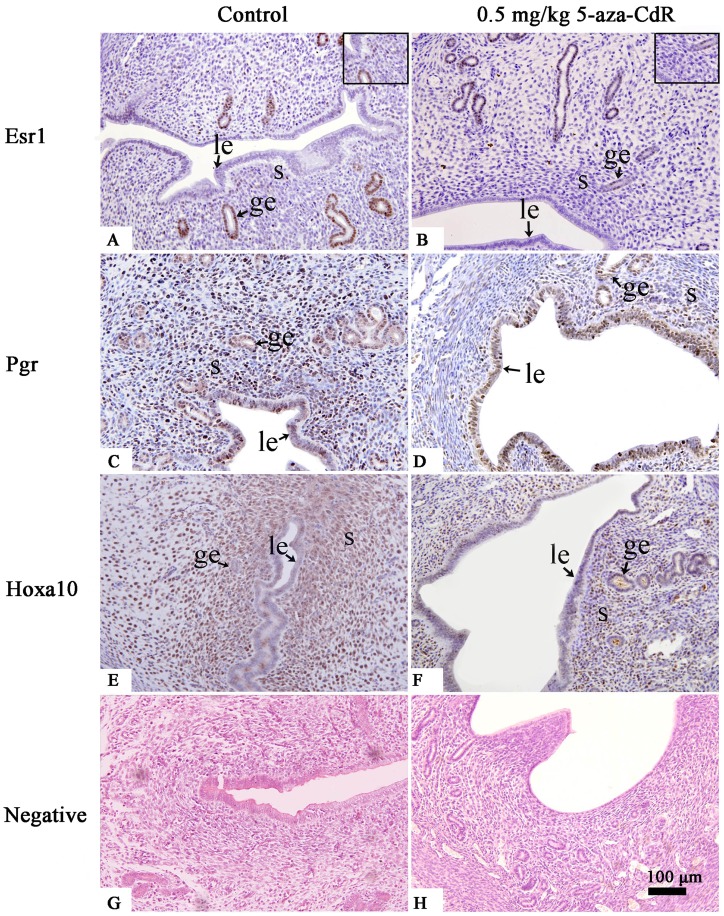
Immunohistochemistry of Esr1 (A, B), Pgr (C, D), and Hoxa10 (E, F) in mouse endometrium on PD5. Mice were treated daily with or without 0.5 mg/kg 5-aza-CdR. The inset boxes in the upper right corners are enlarged region of each figure. le, luminal epithelium; ge, glandular epithelium; s, stroma.

We examined the expression of Esr1, Pgr, and Hoxa10 protein in the endometrium on PD5 using western blot analysis. This revealed that Hoxa10 was significantly repressed at both 0.1 and 0.5 mg/kg 5-aza-CdR, and that Esr1 and Pgr were repressed only at 0.5 mg/kg 5-aza-CdR (Figure 5). Immunohistochemistry revealed that Esr1 was reduced in stroma and glandular epithelium (Figure 6A, B), and Pgr (Figure 6C, D) and Hoxa10 (Figure 6E, F) were reduced in stroma at 0.5 mg/kg 5-aza-CdR. These results are summarized in Table 2.

**Table 2:** Localization of Esr1, Pgr, and Hoxa10 in mouse endometrium following 4 days of 5-aza-2′-deoxycytidine treatment.

Protein	5-aza-CdR (mg/kg)	Stroma	Luminal epithelium	Glandular epithelium
**Esr1**				
	0	+	−	+++
	0.5	−	−	++
**Pgr**				
	0	+++	+++	+++
	0.5	++	+++	+++
**Hoxa10**				
	0	+++	−	−
	0.5	++	−	−

−, no staining; +, weak staining; ++, moderate staining; and +++, strong staining.

## Discussion

It has been shown that intrauterine insult during mid- to late-gestation using the anti-cancer agent and cytidine analog 5-aza-CdR causes temporally related defects in the developing mouse [3], [30]. Our results showed a significantly decreased number of embryo implantation sites on PD5 in mice treated daily with 0.5 mg/kg 5-aza-CdR on PD1–4. Further results showed that the proliferation of endometrial stromal cells was significantly reduced and decidualization was impaired in these mice. Endometrial stromal cell proliferation and differentiation are key processes for successful implantation [31]. Hence, our study indicates that the cytotoxic effects of 5-aza-CdR on embryo implantation might be associated with reduced stromal cell proliferation and differentiation (decidualization). This cytotoxicity of 5-aza-CdR results from its capacity to cause DNA damage, as 5-aza-CdR is a nucleoside analog that can be incorporated into the DNA backbone, which may in turn induce formation of a covalent adduct between the 5-aza-CdR molecule and DNMTs [1].

In addition to its cytotoxic function, 5-aza-CdR demethylates DNA and thereby alters gene expression through DNMT activity, and has been widely used to demonstrate how DNA methylation affects biological processes previously thought to be regulated primarily by genetic factors [1], [7], [11], [12]. The present results show that 5-aza-CdR affected Dnmt expression in a time- and dose-dependent manner in mouse endometrium. Moreover, repression of Dnmt in the endometrium was found to be cell specific. Therefore, it is possible that one or more critical methylation factors may be involved in regulating the cellular response to 5-aza-CdR treatment, and these may vary by cell type in endometrium. Previous studies have shown a significant role for DNA methylation in regulating endometrial changes associated with implantation. Relatively high levels of *DNMT1*, *DNMT3A*, and *DNMT3B* expression are seen in the proliferative phase of the human endometrium, with lower expression in the second secretory phase; this expression pattern is essential for human endometrial changes throughout the reproductive cycle [18], [19]. Recently we found that expression of mouse Dnmt1, Dnmt3a, and Dnmt3b was decreased during the receptive phase compared with the prereceptive phase, and that implantation sites showed significantly lower levels of Dnmt3a mRNA and protein [32]. These observations provide further support for the role of DNA methylation in endometrial changes during embryo implantation. Thus, we suggest that the effect of 5-aza-CdR on implantation might be associated with DNA demethylation through repression of Dnmt expression.

*Esra*, *Pgr*, and *Hoxa10* are essential genes controlling uterine receptivity and decidualization, and their aberrant expression results in implantation defects in mammals [33]–[37]. We found repressed expression of Esr1, Pgr, and Hoxa10 and subsequent defective uterine decidualization in 5-aza-CdR-treated mice, which may explain the implantation failure. Promoter hypermethylation plays a major role in repressing *ESR1* [38]–[40] and *PGR* [41]–[45] expression in human tumors. The *HOXA10* promoter is also susceptible to methylation in the endometria of women wearing intrauterine devices [46], in patients with ovarian cancer [47] or endometriosis [48], and in the uteri of mice exposed to diethylstilbestrol [49]. In general, DNA methylation blocks gene expression, whereas demethylation (e.g., with 5-aza-CdR) activates gene expression. Treatment with 5-aza-CdR reactivated the expression of *ESR1*, *PGR*, and *HOXA10* from their repressed state in the above studies [38]–[49]. However, we found that Esr1, Pgr, and Hoxa10 had reduced expression with hypomethylated promoters after treatment with 5-aza-CdR. This finding suggests that 5-aza-CdR plays diverse roles in cells, including demethylating functions as well as methylation-independent functions [50]. Our results agree well with previous findings that 5-aza-CdR may inhibit gene expression in a methylation-independent manner. *Cx31*, *Cx43*, *Cx45*, *Cdh1*, and *Ctnnb1* are all repressed by 5-aza-CdR in preimplantation embryos [9]. 5-Aza-CdR decreases expression of *MDR1* mRNA in K562/ADM cells [13]. In addition, microchip assays revealed a large number of genes downregulated in human endometrial stromal cells [25] and different cancer cells following 5-aza-CdR treatment [14]–[17].

The mechanism by which 5-aza-CdR decreases gene expression is still unknown, and few studies have addressed this question. Stability of estrogen receptor mRNA is decreased through modulation of HuR (Hu Antigen R) in ER-positive MCF7 cells following 5-aza-CdR treatment [51], which might explain why Esr1 was suppressed by 5-aza-CdR. It has been also found that 5-aza-CdR incorporates into DNA; this may inhibit template function and chain elongation [52], inhibit DNA polymerase α in opposition to the normal substrate deoxycytidine 5′-triphosphate [53], and induce DNA damage in a dose-dependent manner [50]. In addition, the 5-aza-CdR analog 5-aza-cytidine (5-aza-C) induces formation of DNMT-DNA adducts at the DNA replication fork *in vivo* [54], [55]. We suggest that 5-aza-CdR-induced DNMT-DNA adducts may affect DNA replication and expression of Esr1, Pgr, and Hoxa10 as well.

In conclusion, we showed that the effects of 5-aza-CdR on embryo implantation may be due to both cytotoxicity in stromal cell proliferation and differentiation. 5-aza-CdR may interfere with endometrial expression of Dnmts and genes vital for implantation, thereby leading to significant inhibition of embryo implantation.

## Materials and Methods

### Ethics Statement

All experiments involving animals were approved by the Ethics Committee of Chongqing Medical University (Permit Number: 20110016).

### Animals

Kunming mice were purchased from Laboratory Animal Centre, Chongqing Medical University and were housed in separate cages with a 12-h light/dark cycle. Female mice (8–10 wks, 20–24 g) were mated with fertile or vasectomized male mice and checked for vaginal plugs at 0700–0800 the next morning; the presence of a vaginal plug was used to designate day 1 of pregnancy (D1) or pseudo-pregnancy (PD1).

**Table 3:** Primers for bisulfite-sequencing PCR.

Gene	Primer sets	Accession number^a^
***Esr1***	Forward 5′ GGGAGGGGTTGTTAAGTGTT 3′	NM_007956.4
	Reverse 5′ CCCAAAACCCTCTCCATAA 3′	
***Pgr***	Forward 5′ GAGAATTTAGGGAGTTATAGAGATTG 3′	NM_008829.2
	Reverse 5′ CG(A)TAAAATCTCCACCTCCTA 3′	
***Hoxa10***	Forward 5′ TATGGGAGTATTTAAGGTTGGTTG 3′	NM_008263.3
	Reverse 5′ CATTTCTTATACAAAACATACTAAATAC 3′	

a Genebank accession number.

### Experimental Design

Control pregnant mice (*n* = 18) served as embryo donors. Induced pseudo-pregnant mice were randomly assigned to four treatment groups (*n* = 24 each) that received daily intraperitoneal injections [3] of sterile isotonic saline (control) or 5-aza-CdR (0.1 or 0.5 mg/kg; Sigma, St. Louis, MO, USA). Treatment began on PD1 (pseudo-pregnant day 1, first day of the presence of a vaginal plug) and continued for 2 or 4 days to ensure the continual effect of the drug, as it has a short half-life *in vivo*. Six mice from each treatment group received embryo transfer on PD4; each mouse received ten embryos with normal appearance from control pregnant mice. To minimize the effects of 5-aza-CdR on preimplanted embryos, embryo transfer was performed 2 hr after the last 5-aza-CdR treatment on PD4. Twelve pseudo-pregnant mice from each treatment group were sacrificed for tissue collection on PD3 and PD5 (*n* = 6 each). The remaining six female mice were used for artificial decidualization, and 10 µl of sesame oil was injected into each horn in the morning of PD4. Implantation sites were counterstained by intravenous injection with Chicago Blue B solution, 15 min prior to sacrifice.

### Western Blot Analysis

Fragments of endometrial tissues were obtained by finely scraping the luminal wall of the uterus of pseudo-pregnant mice on PD5. Endometrial tissues (200 mg) were weighed and homogenized in cell lysis buffer (Beyotime, Shanghai, China). Protein aliquots (30 µg) were mixed with SDS-sample loading buffer and heated at 100°C for 10 min. Protein was separated by 10% (w/v) SDS-PAGE and electrotransferred onto nitrocellulose membranes (Bio-Rad Laboratories, Foster City, CA, USA), which were incubated in blocking buffer (20 mM Tris, pH 7.6, 137 mM NaCl, 0.05% (v/v) Tween 20, and 10% nonfat dry milk) for 2 h and then incubated at 4°C overnight with primary polyclonal rabbit antibodies against Dnmt1, Dnmt3a, Dnmt3b (Santa Cruz Biotechnology, Santa Cruz, CA, USA; all 1∶400) and Pgr (Abcam, San Francisco, CA, USA; 1∶500), or monoclonal antibodies against Esr1 and β-actin (Abcam; both 1∶1000), or goat antibody against Hoxa10 (Santa Cruz, 1∶200). After three washes with blocking buffer, membranes were incubated with horseradish peroxidase-conjugated rabbit anti-goat or goat anti-rabbit IgG secondary antibody (both 1∶200, Zhongshan Golden Bridge, Beijing, China). After additional washes, immunoreactive bands were visualized using enhanced chemiluminescence reagents (Beyotime). Densitometry was performed using Quantity One v4.4.0 (Bio-Rad Laboratories) with β-actin as a loading control.

### Histologic and Immunohistochemical Staining

Uterine tissues were fixed in 4% paraformaldehyde in phosphate-buffered saline (PBS) for 12 hr, air-dried, and embedded in paraffin. Sections (4 µm thick) were cut, mounted on slides, deparaffinized with a graded series of xylene, and rehydrated in a descending graded alcohol series. Sections were immersed in 0.1 M citrate buffer and boiled for 15 min in a microwave for antibody retrieval. Once the slides had cooled at room temperature, they were washed for 10 min in TBST (Tris-buffered saline, pH 7.6, with 1% (w/v) Tween 20) and rinsed in distilled H2O. Sections were blocked in 3% H2O2/methanol for 10 min at room temperature, incubated with blocking solution containing 10% normal goat or rabbit serum in TBST for 30 min at room temperature, then incubated with primary antibodies for Dnmt1, Dnmt 3a, Dnmt 3b, Hoxa10 (Santa Cruz Biotechnology; 1∶100, 1∶200, 1∶100, and 1∶50, respectively), Pgr and Esr1 (Abcam; 1∶200 and 1∶300, respectively) or Ki67 (Millipore, Temecula, CA, USA; 1∶300) overnight at 4°C. Sections were washed three times with TBST, then incubated with secondary antibody (biotinylated goat anti-rabbit or rabbit anti-goat IgG, Zhongshan Goldenbridge, 1∶200) for 1 h at room temperature. After one wash, sections were analyzed using the StrepABC horseradish peroxidase kit (Beyotime). Negative controls were created in parallel by replacing the primary antibody with PBS. All sections were lightly counterstained with hematoxylin and eosin (Beyotime). Image-Pro Plus (Media Cybernetics, Bethesda, MD, USA) was used to quantify images. The intensity of Dnmt staining was classified as no staining (–), weak staining (+), moderate staining (++), or strong staining (+++).

### Bisulfite Sequencing PCR (BSP)

Genomic DNA was extracted from frozen endometrial tissue of control and 5-aza-CdR-treated mice (*n* = 3 each) using a standard DNeasy Blood and Tissue Kit (Qiagen, Valencia, CA, USA). Genomic DNA (1 mg) was modified by bisulfite to convert unmethylated cytosines to uracil using the Methylamp DNA Modification Kit (Epigentek, Brooklyn, NY, USA). Bisulfite-modified DNA was dissolved in 20 µl water and stored at −80°C. The flanking regions () of *Esr1*, *Pgr*, and *Hoxa10*, were sequenced by BSP using primers listed in Table 3. PCR was carried out at 98°C for 4 min, followed by 20 cycles of 94°C for 45 s, 66°C (−0.5°C each cycle) for 45 s, and 72°C for 1 min, with a final incubation at 72°C for 7 min. The resulting first-stage PCR product was amplified using 20 additional cycles of 94°C for 45 s, 56°C for 45 s, and 72°C for 1 min, with a final incubation at 72°C for 8 min. PCR products were purified using the QIAquick PCR purification kit (Qiagen) and sequenced by Sangon Biotechnology.

### Alkaline Phosphatase Staining

Isolated fresh uterine tissues on PD6 were fixed in 2% paraformaldehyde in PBS for 20 min and then cryoprotected in 30% sucrose/PBS for 18 hr at 4°C. Sections (4 µm thick) were cut and embedded in OCT compound (Sakura Finetek, Torrance, CA, USA), and stained with 5-bromo-4-chloro-3-indolyl phosphate and nitro blue tetrazolium chloride (both from Beyotime), and counterstained with Nuclear Fast Red (Beyotime).

### Statistical Analysis

Comparisons between two or more groups were made using Fisher’s exact test, *t* test, and analysis of variance. The CpG methylation rates of genes were analyzed by the Fisher’s exact test. Pearson’s or Spearman’s rank correlation coefficient was used for evaluating correlation between two variables. To see whether 5-aza-CdR treatment or other possible factors were responsible for the change in implantation before and after the treatment, a multiple linear regression model was used. A *p* value <0.05 was considered statistically significant.

## Supporting Information

Figure S1 **Schematic diagram of flanking regions of bisulfite-sequenced genes.** Upper panels: UCSC genome browser view of gene structures for *Esr1* (A), *Pgr* (B), and *Hoxa10* (C). Lower panels: Sequences of promoter regions containing CpG sites (bold). All sequences are 5′ to 3′. Exons are in upper case, everything else in lower case. Arrows indicate transcription start sites.
(TIF)
