# Supplementary figures and images for: 5-Aza-2′-deoxycytidine Leads to Reduced Embryo Implantation and Reduced Expression of DNA Methyltransferases and Essential Endometrial Genes
Source: PLoS One. 2012 Sep 28;7(9):e45364. doi: 10.1371/journal.pone.0045364 (PMC3460940; doi:10.1371/journal.pone.0045364)

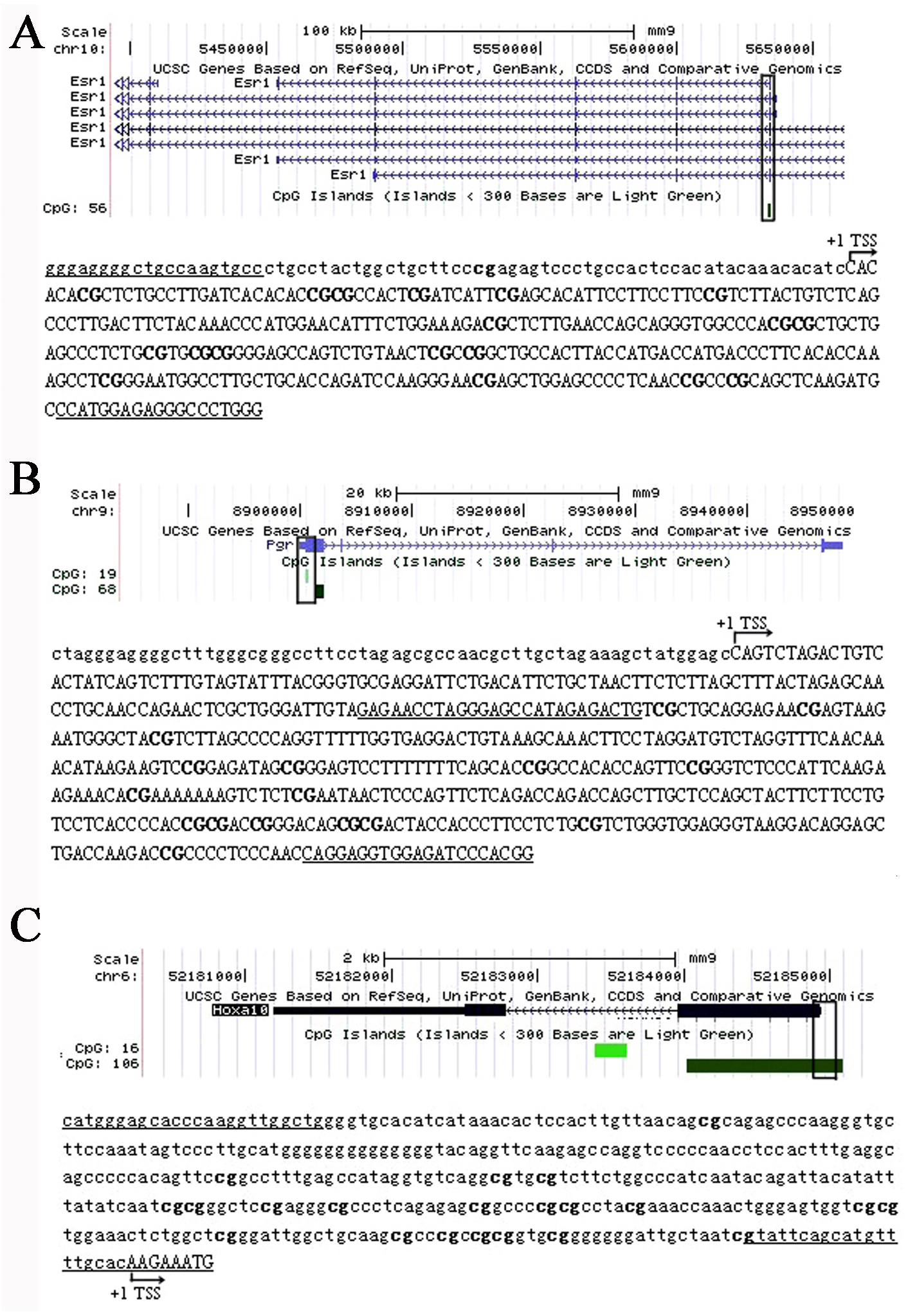

Supplement: Figure S1 — Schematic diagram of flanking regions of bisulfite-sequenced genes. Upper panels: UCSC genome browser view of gene structures for Esr1 (A), Pgr (B), and Hoxa10 (C). Lower panels: Sequences of promoter regions containing CpG sites (bold). All sequences are 5′ to 3′. Exons are in upper case, everything else in lower case. Arrows indicate transcription start sites. (TIF) [file pone.0045364.s001.tif]
